# Feasibility, acceptability and preliminary impact on diet quality of the Healthy Weight for Life™ + medical nutrition therapy; a randomised controlled pilot study

**DOI:** 10.1186/s12966-026-01885-1

**Published:** 2026-03-10

**Authors:** Erin D Clarke, Georgina M Britton, Nicole Hedditch, Ryan Gallagher, Hailey Donnelly, Clare E Collins

**Affiliations:** 1 School of Health Sciences, College of Health, Medicine and Wellbeing, University of Newcastle, Callaghan, NSW 2308 Australia; 2 Food and Nutrition Research Program, Hunter Medical Research Institute, New Lambton Heights, NSW 2305 Australia; 3 Honeysuckle Health Pty Ltd, Newcastle, NSW 2300 Australia

**Keywords:** Diet quality, Weight management, Medical nutrition therapy

## Abstract

**Background:**

Poor diet quality has been associated with greater risk of people developing overweight and obesity, type 2 diabetes, cardiovascular disease, and osteoarthritis. For each of these conditions, weight management is recommended in respective guidelines. Current weight management interventions, such as the Healthy Weight for Life™ (HWFL) program that uses meal replacements, have not been assessed for impact on diet quality. Further, the role of a dietitian providing medical nutrition therapy (MNT) in conjunction with the HWFL program has not been evaluated. MNT is an evidence-based approach where dietary interventions are tailored to the individuals needs, medical history, lifestyle, and dietary preferences. Therefore, the primary aim of this pilot study is to evaluate the feasibility, acceptability and preliminary impact of adding personalised (MNT) consultations to the HWFL program on diet quality. Secondary outcomes include weight and osteoarthritis scores .

**Methods:**

An 18-week randomised control trial was undertaken, with eligible HWFL program participants randomised to either usual care arm (HWFL program) or HWFL + MNT. A food frequency questionnaire was used to assess diet quality (% energy from nutrient-dense core food) and provide intervention participants with personalised feedback on food and nutrient adequacy of their dietary patterns. Weight and knee osteoarthritis outcomes using the hip and knee osteoarthritis outcome scores, were self-reported and used to assess outcomes. Project acceptability was assessed by process evaluation questionnaire. An intention-to-treat analysis was undertaken using generalised linear mixed models with post-estimations reported as mean (95% CI).

**Results:**

Forty participants (75% female, 62 ± 10years, 78% osteoarthritis) enrolled in the study. Baseline diet quality was poor (34% energy from non-core foods). Post-intervention both intervention and usual care groups significantly improved their diet quality and reduced weight, but there were no significant differences between groups. Sub-scores for knee osteoarthritis function significantly improved in the intervention compared to the control group (mean between group difference 17.4, 95% CI 1.6, 33.1), though after correcting for multiple testing this was no longer significant. Overall intervention acceptability was high.

**Conclusion:**

Both the intervention and usual care arms significantly improved diet quality. All intervention participants found MNT was highly acceptable. For those with knee osteoarthritis significant improvements in knee osteoarthritis function was reported in the intervention group. This should be further explored in future interventions.

**Trial registration:**

Australian New Zealand Clinical Trials Registry (ACTRN12623001062617).

**Supplementary Information:**

The online version contains supplementary material available at 10.1186/s12966-026-01885-1.

## Background

Globally, dietary risks, including low intakes of fruit, vegetables, wholegrains and high intakes red meat and energy-dense, nutrient-poor foods, are the second and third greatest risk factor for all-cause mortality in females and males, respectively [1]. In 2019, poor diet quality accounted for almost 15% of global deaths [1]. Further, poor diet quality has been associated with greater risk of people developing overweight and obesity [2, 3], type 2 diabetes (T2DM) [4], cardiovascular disease (CVD) [5], and osteoarthritis [6]. Worldwide, approximately 528 million people live with osteoarthritis [7]. Osteoarthritis is a chronic, progressive joint disorder characterised by decline in bone joint cartilage, with inflammation and reduction of smooth movements causing pain, aching and/or stiffness [6]. For each of these conditions, especially osteoarthritis, weight management, including dietary interventions and/or referral to an Accredited Practising Dietitian (APD), are recommended in the respective management guidelines, including to assist people with weight management [8–10].

Medical nutrition therapy (MNT) is an evidence-based approach utilising the Nutrition Care Process (Nutrition assessment, diagnosis, intervention, and monitoring and evaluation) [11]. The aim of MNT is to prevent, delay progression or manage a condition [11]. MNT provides personalised advice tailored to the individuals needs, medical history, lifestyle, and dietary preference, differing from other dietary interventions that provide general population dietary advice [11]. MNT provided by an APD has been shown to significantly improve health outcomes for people with overweight and obesity ((Weighted mean differences)WMD weight loss: -1.03 kg [95% CI:-1.40; -0.66]) [12, 13]; T2DM (WMD HbA1c: -0.45% [95% CI: -0.36%; -0.53%]) [12, 14]; and CVD (WMD systolic blood pressure: −2.11mmHg [95% CI: −3.97, −0.25 mmHg] and WMD diastolic blood pressure: −1.46mmHg [95% CI: −2.67, −0.25 mmHg]) [12, 15]). For people with osteoarthritis, referral to a nutritionist for weight management resulted in a significantly higher number of eligible individuals for total joint arthroplasty surgery compared to those who were not referred [16].

An individual’s engagement in managing their own health influences their health outcomes, with individuals who engage more in their own healthcare shown to have better health outcomes when managing chronic diseases [17–19]. Assessing an individual’s engagement in managing their own health can also be an indicator of both engagement and success when undertaking an intervention [20, 21]. Further, increased patient activation has been associated with better quality of life compared to those with lower level of activation [22]. While there is an association between patient activation, health and quality of life, lesser is known about the relationship with dietary intake. Therefore, it is important to assess the impact of an intervention on patient activation and health outcomes, including dietary interventions, providing insights into intervention success, longer-term maintenance and the effectiveness of interventions, as well as the role of an APD. While this study focuses on patient activation, it is important to acknowledge that obesity is complex and the factors impacting weight management including negative emotional states, genetic disposition, physical environment including work environments, and prescription psychiatric medications [23].

The Healthy Weight for Life™ (HWFL) program (Prima Health Solutions, a subsidiary of Honeysuckle Health, Brookvale, NSW, Australia) is an 18-week telehealth program that consist of three phases, refer to Table 1. HWFL provides participants with clinical support, education, tools, and resources needed to help them achieve both weight reduction, health, and lifestyle goals. There are four versions of the HWFL program: the Essentials Plus, Heart Health, Osteoarthritis and Type 2 Diabetes. All programs use the same structure with additional focus on health specifics relevant to the program they are enrolled in [24].

**Table 1:** Healthy Weight for Life™ program outline

Phase Number (Duration)	Outline of Program Intervention
Phase one (Weeks 1–6)	• Replace 2x meals/day with KicStart (Prima Health Solutions, Brookvale, NSW, Australia) formulated very low energy diet (VLED) meal replacement product. • 1x energy-controlled meal and snacks from an energy-controlled plan. • Includes low energy ‘free foods’ such as salad, vegetables and berries. • Initiating and progressively building up daily physical activity such as walking for 3 × 10 min blocks / day (within personal abilities). • 6 targeted phase 1 educational modules.
Phase two (Weeks 7–12)	• Replace 1x meal/day with KicStart (Prima Health Solutions, Brookvale, NSW, Australia) formulated VLED meal replacement product. • 2x energy-controlled meals and snacks. • Includes low energy ‘free foods’ such as salad, vegetables and berries. • 6 targeted phase 2 educational modules.
Phase three (Weeks 13–18)	• Maintenance phase. • 3x energy-controlled meals and snacks per day. • Use of VLED intermittently as required. • Includes low energy ‘free foods’ such as salad, vegetables and berries. • Maintain daily physical activity such as walking for 30 min. • 6 targeted phase 3 educational modules.

Traditional weight management advice involves prescriptive calorie restriction and generic dietary advice [25]. The addition of MNT to the existing HWFL program differs from traditional weight management approaches through integration of personalised MNT, facilitated by an APD, which includes tailored dietary advice and SMART goal setting that considers the individuals barriers and facilitators to dietary behaviour change [11]. Providing personalised behaviour-focused dietary advice and counselling is imperative to achieve long-term weight maintenance, as weight regain is common in people living with obesity. While there is evidence demonstrating very low calorie diets and intermittent fasting can achieve weight loss in the short term, adherence can be difficult [25]. Contrastingly, MNT is an evidence-based effective strategy recommended in clinical guidelines for people living with overweight or obesity to improve quality of life, weight management, and cardiometabolic outcomes [26]. Effective weight management is client-centred with the individual remaining engaged in long-term healthy behaviours [26]. The addition of MNT to the HWFL program has the potential to support sustainable lifestyle changes, providing individualised advice and addressing barriers to change, making it a cornerstone for long-term sustained weight management [26].

Therefore, the primary aims of this pilot study was to assess the feasibility, acceptability and preliminary impact on diet quality of adding personalised MNT consultations from an APD to the HWFL programs (Essentials Plus, Heart Health, Osteoarthritis and Type 2 Diabetes). Secondary aims include assessing the difference in anthropometric measures, quality of life, osteoarthritis, patient activation between intervention and usual care groups.

## Methods

Participants completed an 18-week parallel randomised controlled trial where participants were randomised to the HWFL program (usual care arm) or the HWFL + MNT (intervention arm). Participants who enrolled in any of the HWFL programs between January – April 2024 were invited to participate in the intervention. Data was collected on diet, anthropometric measures, quality of life, osteoarthritis outcomes, patient activation and participant acceptability. Ethics approval was received from the University of Newcastle Human Research Ethics Committee (H-2023-0206) and the trial was registered prospectively with the Australian New Zealand Clinical Trials Registry (ACTRN12623001062617). All participants provided written informed consent. Reporting was conducted using the CONSORT extension for randomised pilot and feasibility studies (Supplementary file 1) [27].

### Eligibility criteria

Eligible participants were adults (18 + years of age) who were eligible for the HWFL program (including both private health insurance funded and self-funded participants), Table 2. Participants where excluded from the study if:

- They were involved in the HWFL program however did not consent to being involved in the research project or
- They did not complete all of their baseline questionnaires or
- They were not eligible for the HWFL program.

**Table 2:** Healthy Weight for Life™ program eligibility criteria

All program eligibility criteria	Additional specific program eligibility
BMI ≥ 28 kg/m^2^	*Heart -* Being treated for, or have a history of, at least one of the following: high blood pressure, high cholesterol, angioplasty or stent, heart attack/stroke, coronary bypass surgery, cardiac arrhythmia/angina, obstructive sleep apnoea
A relevant hospital policy with participating private health funds that has been held for at least 12 months	*Diabetes–* A diagnosis of type 2 diabetes mellitus
Willingness to provide progress data over the course of the 18-week program	*Osteoarthritis–* A diagnosis of symptomatic knee or hip osteoarthritis *
Not previously participated in a HWFL program funded by the individual’s private health insurance	*Essentials Plus–* Have one or more existing chronic health condition that is not eligible for heart, diabetes, and osteoarthritis HWFL

* All programs use the same HWFL phases, except for the Osteoarthritis program which includes an additional exercise education component.

### Intervention

#### Healthy Weight for Life (usual care)

The HWFL program is run by Prima Health Solutions Pty Ltd (a subsidiary of Honeysuckle Health), an Australian allied healthcare and technology company. The 18-week, 3 phase HWFL program provides participants with the clinical support, education, tools and resources need to help them achieve their weight loss and lifestyle modification goals. Patients can enrol in the HWFL program if they are members of eligible health funds at no cost, or participants who are not members of a partnering health fund can complete the program for a fee.

Program participants are offered clinical support from allied health and nursing support staff via phone, SMS, email, private digital message board and mail throughout the program between Weeks 1 and 18 (Supplementary file 2). Printed and online education is provided to all participants covering topics including goal setting, mindful eating, healthy eating, portion planning, recipes, increasing physical activity, maintaining motivation, sleep hygiene, overcoming weight loss plateaus, and long-term maintenance strategies.

**Fig. 1:**
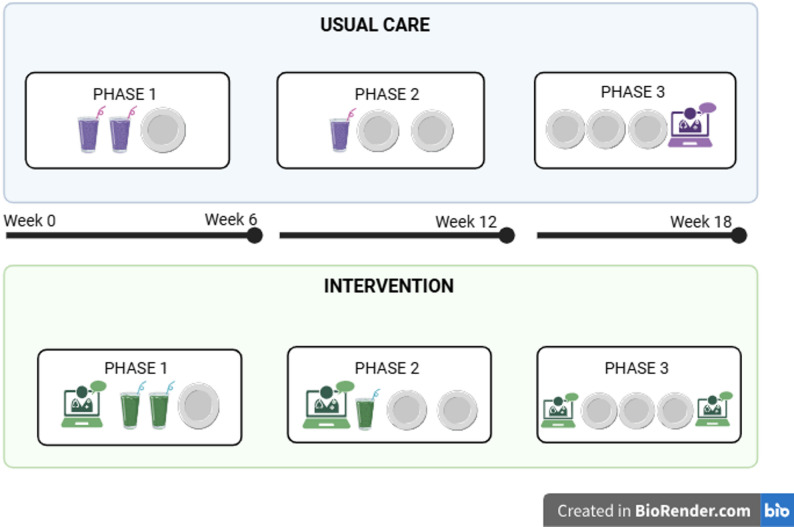
Healthy Weight for Life™ program summary. Created in https://BioRender.com

The HWFL program is structured into three, six-week phases, Fig. 1. Participants are provided with formulated meal replacement options (e.g. shakes and soups) which they titrate across the program.

In addition to the HWFL program, the usual care arm was offered one optional 30-minute MNT consult with an Accredited Practising Dietitian (APD) at the end of the 18-week program.

#### Healthy Weight for Life + medical nutrition therapy (intervention)

Intervention participants received the HWFL program as usual and in addition received four telehealth personalised MNT consultations from an APD, totalling 1.5 h of MNT over 18-weeks. Consultations were held between Week 0 (before commencing), Week 7 (first week of Phase 2), Week 13 (first week of Phase 3) and Week 18 (at the end of Phase 3), or within the first few weeks of starting each phase. The initial consult was 30-minutes, and each consecutive consult was 20-minutes (Fig. 1). These consultation times align with Australian Medicare rebate time periods and while initial dietitian consults can take approximately 60 min and review consults can take 30 min [28], all demographic and dietary data was collected prior to the consults, reducing the time required.

The MNT consults were constructed around participants results from the Australian Eating Survey- Heart (AES-Heart) report (Supplementary File 3) [29] and their answers to the Personalised Nutrition Questionnaire (PNQ) (see data collection methods for further information) [30]. This involved reviewing results from participant’s AES-Heart report and discussing goals related to components their nutrition report and what the participant wanted to prioritise. Discussions were also held with the participant about their self-identified barriers to making changes to their diet. Using this data participants were guided to set 1–3 nutrition related goals and be provided with resources e.g. education, recipe ideas etc. that will help them to make these dietary changes and reach their goals. Follow up consultations focused on participant’s progress with achieving their goals, and with adjustment made as required.

Medical nutrition therapy consults were conducted through a secure online platform (Coviu, Coviu Global Pty Ltd, New South Wales, Australia). Appointments were booked through Halaxy (Halaxy Pty Ltd, Victoria, Australia), a commonly used practice software for Australian APDs. All consultation notes from the APD were completed on REDCap [31, 32]. At the completion of the consultation APDs documented a short summary communication regarding the participant in the internationally recognised ISBAR (Introduction, Situation, Background, Assessment, Recommendation) format. This summary facilitated handover and allowed for continuity in care, escalation of clinical or administrative involvement in the HWFL program and to maintain positive customer interactions.

At the end of each appointment the dietitian emailed the participant an appointment summary highlighting the key nutrition issues, goals set and copies/links to any resources discussed.

### Randomisation

Participants were randomised 1:1 to the usual care arm or the intervention arm after they had completed all baseline surveys. The randomisation code was created in blocks of six and managed by someone external to the project to allow the project manager (EDC) to be blinded until after the completion of baseline surveys [33].

### Sample size

As this was a pilot and feasibility study a sample size calculation was not required. However, a target of 40 participants was deemed appropriate based on our prior experience in recruitment in feasibility trials as well as the fundings limited timeframe.

### Data collection

#### Personalised nutrition questionnaire

Participants completed the online PNQ prior to their initial consultation [30]. This questionnaire was reviewed prior to the consult by the dietitian and used to help identify barriers and facilitators relating to the participants capability, opportunity and motivation that the individual believes to be impacting on the ability to eat healthily. The theoretical framework underpinning the dietetic intervention was Michie’s behaviour change paradigm which incorporates previously identified behaviour change intervention characteristics and policies into Capability, Opportunity, Motivation – Behaviour (COM-B) system [34]. The PNQ included three questions, one for each of the three COM-B domains, with the question for capability have nine options, opportunity question having four options, and the question for motivation having six options. Response options encompassed knowledge, skills, time/access, support, beliefs, and planning.

During the consultations, the APD was able to use the participant’s PNQ responses and appropriate resources to direct discussion around goal setting, nutrition interventions, and behaviour change. The PNQ resource library included references to the already supplied HWFL program resources to ensure consistency in messages across the HWFL program and MNT consultations.

#### Patient Activation Measure (PAM)

The PAM-10 is a 10-item measure that categorises participants according to their knowledge, skills, and confidence in managing their own healthcare [35]. Participants completed this questionnaire at baseline and week-18. Higher scores align with greater knowledge, skills and confidence in managing their own healthcare.

#### Self-reported measures collected as part of the HWFL program

Several measures are collected as part of the HWFL program, these included self-reported height (cm), weight (kg), waist circumference (cm), and quality of life using the 12-item Short Form Health Survey (SF-12) [36]. For participants in the osteoarthritis program, osteoarthritis measures using the Knee Injury and Osteoarthritis Outcome Score (KOOS) [37] or the Hip Dysfunction and Osteoarthritis Outcome Score (HOOS) [38] were also collected.

Total SF-12 scores were calculated as the sum of all points, with a maximum score of 47 possible. Whereas physical scores were calculated as the sum of the six questions on physical health (maximum score 20) and mental scores as the sum of the six questions on emotional health (maximum score 27). Higher scores are associated with more optimal health.

KOOS and HOOS scores were calculated for subscales: Pain, Symptoms, Function, Sport and Recreation Function, and Quality of Life. Each subscale was scored between 0 and 100 and, with lower scores representing more extreme knee or hip problems [37, 38].

Weight and waist circumference were collected at the end of each phase of the program, whereas quality of life and osteoarthritis measures were collected at the start and end of the program.

### Process evaluation

Participants were asked to complete a 12-item process evaluation at the end of the project. This included a mix of Likert Scale and open-ended questions and was designed specifically for the current intervention.

The process evaluation asked participants how beneficial they thought the consults were, did they think they were long enough, and would they prefer more or less dietitian consults in the future.

### Statistical analysis

All statistics were carried out in Stata/IC 14.2 (StataCorp, College Station, TX, USA). Normality was assessed for primary outcome using Shapiro Wilks. Descriptive statistics were conducted and reported as mean ± SD or n (%) unless otherwise specified. Baseline differences were assessed by randomisation arm using t-test for continuous variables or chi2 for categorical variables. Missingness of data for the primary outcome was checked.

An intention to treat analysis was undertaken using generalised linear mixed models with post-estimations reported as mean (95% CI). Predictors used in the models included time (categorial using baseline and 18-weeks), treatment group (intervention or usual care), and an interaction for time by treatment group using a maximum likelihood estimation to assess pre-post intervention differences between treatment groups. All models were checked for their fit and whether they met assumptions. For alcohol intake the original model violated normality assumptions due to skewed distribution, therefore alcohol was log transformed and used in the mixed model. Due to a small sample of participants with hip osteoarthritis, modelling could not be used to report change pre-post intervention for hip osteoarthritis outcomes. Standardised effect sizes were calculated by dividing the model coefficients by the baseline standard deviation and classified as small (*d* = 0.2), medium (*d* = 0.5) or large (*d* = 0.8) [39]. Significance was set at 0.05, however to account for multiple testing a Bonferroni corrected p-value threshold was set at 0.002.

### Dietary intake

Dietary intake was measured using the AES-Heart. The AES-Heart consists of 177 items and uses the AUSNUT2011-13 food and nutrient database to provide qualitative and quantitative feedback on food and nutrient intakes compared to Australian Dietary Guidelines [40], Heart Foundation Recommendations [41] and Nutrient Reference Values [42]. This is an extended version of the AES and captures additional details related to food specific to heart health dietary recommendations regarding intake of saturated fat, sodium, omega-3 fat food sources, fruits, vegetables, and which are foods and nutrients that are known to impact blood lipids. This is an online dietary assessment tool that provides immediate personalised feedback on eating habits related to heart health. The AES-Heart has previously been validated against objective red blood cell membrane fatty acid concentrations and may be used to assess intake over the previous 3–6 months [29]. This questionnaire was completed by participants at baseline and week-18. The personalised diet report generated was used to provide participants with personalised dietary advice. An example report can be found in Supplementary File 3. Each personalised feedback report provides information on the proportion of energy from core and non-core foods, food group serve intakes, and comparison of macro- and micro-nutrient intakes against national nutrient recommendations [40–42].

## Results

### Baseline characteristics

**Fig. 2:**
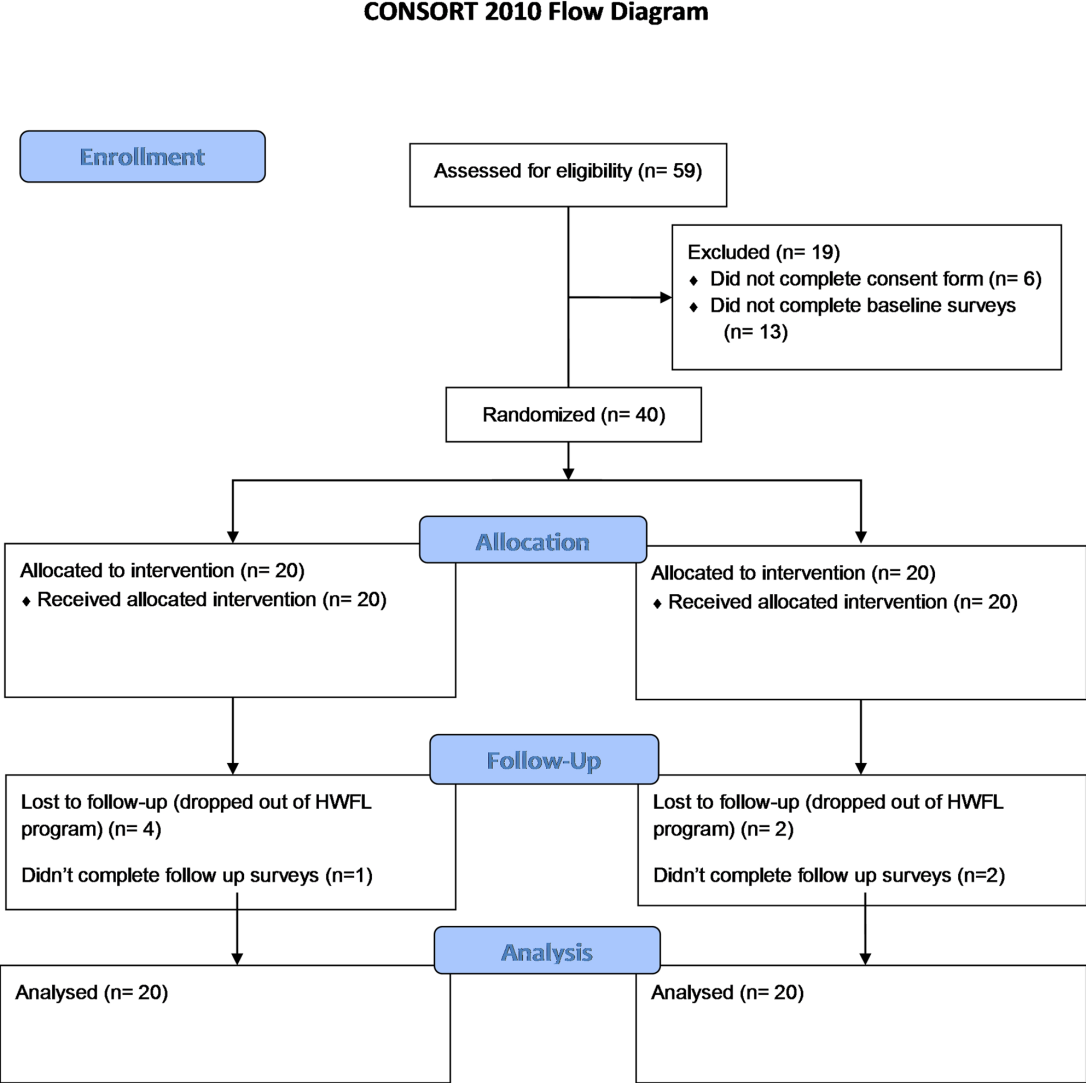
CONSORT diagram Healthy Weight for Life + medical nutrition therapy

In total 59 people expressed interest in the program and 53 provided consented. Of these 40 completed baseline questionnaires and were randomised (CONSORT flow chart, Fig. 2). Participants were primarily female (75%), aged 62.0 ± 9.7 years and enrolled in the Osteoarthritis program (77.5%). Of the participants with osteoarthritis, 24 participants had knee osteoarthritis and 7 with hip. Other than osteoarthritis, the most common comorbidities were high cholesterol and type 2 diabetes. The only significant differences between the intervention and usual care group at baseline were for the participants (*n* = 7) with hip osteoarthritis, which showed that the HOOS sub-scale scores for symptoms, pain and function which indicated that at baseline the usual care group had significantly worse symptoms, however the groups were not equal with only two participants in the usual care group Table 3. No harms or unintended effects were reported.

There were no statistically significant differences in baseline dietary intakes between groups, with overall intakes of poor nutritional quality, including approximately a third of total energy derived from energy-dense, nutrient-poor, non-core foods, Supplementary File 4.

At program completion there were 6 dropouts from the HWFL program (15%), although three completed the study follow up surveys and one completed the AES-Heart. There were no significant differences at baseline between those who completed the study versus those who dropped out for the primary outcome of percentage energy from core foods.

**Table 3:** Baseline characteristics of Healthy Weight for Life + medical nutrition therapy participants

	HWFL + MNT (*n* = 20)	HWFL Usual Care (*n* = 20)	Total Sample (*n* = 40)	Between group differences ^a^
HWFL Program *n*(%)				
Essentials Plus	1 (5)	0 (0)	1 (2.5)	0.67
Osteoarthritis	15 (75)	16 (80)	31 (77.5)	
Heart health	3 (15)	2 (10)	5 (12.5)	
Type 2 diabetes	1 (5)	2 (10)	3 (7.5)	
Self-reported Nutrition-Related Primary Health Conditions n(%) ^b^				
High BP	0 (0)	4 (20)	4 (10)	0.04
High cholesterol	2 (10)	5 (25)	7 (17.5)	0.22
T2DM	2 (10)	3 (15)	5 (12.5)	0.64
Osteoarthritis	11 (55)	14 (70)	25 (62.5)	0.34
GI Conditions ^c^	2 (10)	1 (5)	3 (7.5)	0.56
Reflux	1 (5)	1 (5)	2 (5)	1.00
Atrial fibrillation	4 (20)	0 (0)	4 (10)	0.04
Gallstones	1 (5)	1 (5)	2 (5)	1.00
Fatty liver	1 (5)	0 (0)	1 (2.5)	0.32
Cholecystectomy	0 (0)	2 (10)	2 (5)	0.15
Gout	0 (0)	2 (10)	2 (5)	0.15
CVD	0 (0)	2 (10)	2 (5)	0.15
Sleep apnoea	1 (5)	1 (5)	2 (5)	1.00
Hypothyroidism	1 (5)	0 (0)	1 (2.5)	0.32
Age (years)	61.5 ± 2.5	62.5 ± 1.8	62.0 ± 9.7	0.74
Sex n(%)				
Male	3 (15)	7 (85)	10 (25)	0.14
Female	17 (85)	13 (15)	30 (75)	
Anthropometric measures				
Weight (kg)	99.0 ± 18.0	100.7 ± 16.1	99.9 ± 16.9	0.75
BMI (kg/m^2^)	36.0 ± 6.0	35.4 ± 5.4	35.7 ± 5.6	0.75
Waist circumference (cm)	113.6 ± 13.7	115.6 ± 11.8	114.6 ± 12.6	0.62
Quality of life (SF-12)				
Physical Component (SF-12 PCS) Score range 6–20	13.1 ± 1.1	12.5 ± 1.5	12.8 ± 1.3	0.15
Mental Component (SF-12 MCS) Score range 6–27	19.7 ± 1.6	20.2 ± 2.0	19.9 ± 1.8	0.44
Total SF-12 Score Score range 12–47	32.8 ± 2.1	32.7 ± 2.3	32.7 ± 2.2	0.83
Osteoarthritis measure – Knee Pain (KOOS) n(%)^d^	10 (50)	14 (70)	24 (60)	
Symptoms Score	49.7 ± 20.7	58.9 ± 22.3	55.0 ± 21.7	0.32
Pain Score	58.6 ± 20.9	60.8 ± 20.6	59.9 ± 20.3	0.80
Function Score	56.6 ± 20.2	63.6 ± 23.2	60.7 ± 21.8	0.45
Sport Score	19.0 ± 14.1	24.6 ± 18.7	22.3 ± 16.8	0.43
Quality of Life Score	31.0 ± 17.6	32.9 ± 21.3	32.1 ± 19.5	0.82
Osteoarthritis measure – Hip Pain (HOOS) n(%)^d^	5 (25)	2 (10)	7 (17.5)	
Symptoms Score	77.0 ± 11.0	35.0 ± 14.1	65.0 ± 23.1	0.01
Pain Score	69.8 ± 8.1	37.0 ± 14.1	60.4 ± 18.3	0.01
Function Score	78.8 ± 11.1	34.5 ± 13.4	66.1 ± 24.1	0.01
Sport Score	38.6 ± 33.4	15.5 ± 4.9	32.0 ± 29.5	0.40
Quality of Life Score	57.4 ± 17.4	28.0 ± 39.6	49.0 ± 25.9	0.20
Patient Activation Measure	62.6 ± 2.6	62.7 ± 11.7	62.6 ± 11.6	0.99

^a^ Determined using t-test or Chi2

^b^ Adds to more than 100% as participants could have more than one condition

^c^ Gastrointestinal conditions included diverticulitis, Crohn's disease, IBS as defined by the Healthy Weight for Life team

^d^ Only completed by participants in the Osteoarthritis program

### Process evaluation (acceptability)

Fourteen participants (70%) completed the process evaluation questionnaire post intervention. Overall, responses were positive with majority of participants agreeing or strongly agreeing that the program was beneficial (100%), supportive (92.9%), tailored to their individual needs (92.9%) and complementary to the HWFL program (92.9%) ( Supplementary File 5). Only one participant disagreed with statements, which may be due to their dietitian leaving the program part way through the intervention and hence experiencing a loss of continuity of care.

### Change in primary and secondary outcomes post-intervention

Both intervention and usual care groups significantly increased their mean percentage energy from nutrient-dense core foods, but this was not statistically significantly different between groups, Table 4.

Significant within group differences for the intervention arm but not the usual care group were observed for several dietary outcomes including, reduction in energy, total fat, carbohydrates and alcohol intake and increasing the number of seafood serves. Only the number of serves of vegetables significantly increased in the usual care group but not the intervention group. None of these were statistically significantly different between groups (Table 4).

Other outcomes including weight and waist circumference significantly reduced in both groups. There were also significant improvements in PAM, KOOS symptom, pain, sports and quality of life outcomes in both groups. For those with knee osteoarthritis only KOOS function increased in the intervention arm with this change remaining statistically significant between groups (17.4, 95% CI 1.6, 33.1, *p* = 0.03). Refer to Table 4. However, after adjusting for multiple testing this result was no longer significant.

Overall, most effect sizes were small and favoured the intervention group (*n* = 16/23). The SF-12 Mental outcome was classified as moderate (0.59) and KOOS Function score classified as large (0.80), both favouring the intervention group (Table 4). For the primary outcome (percentage energy from core foods) both the intervention group (effect size = 0.87) and the usual care group (effect size = 0.85) had large within group effect sizes.

**Table 4:** Within and between group changes post intervention for the Healthy Weight for Life™ + medical nutrition therapy intervention

	HWFL + MNT Arm (Change from Baseline)			Usual Care Arm (Change from Baseline)			Mean difference between groups Group#Time			Effect Size
	Mean	95% CI	*p*-value	Mean	95% CI	*p*-value	Mean	95% CI	*p*-value	
DIETARY INTAKE										
% Energy core	12.8	6.0, 19.6	< 0.001	12.5	5.0, 19.9	0.001	0.3	-9.8, 10.4	0.95	0.02
Energy (kJ/day)	-1840.0	-3129.9, -550.0	0.005	-1225.1	-2662.6, 212.5	0.10	-614.9	-2546.3, 1316.6	0.53	-0.19
Protein (g/day)	2.0	-12.4, 16.5	0.78	-4.5	-20.7, 11.7	0.59	6.5	-15.2, 28.2	0.56	0.15
Fat (g/day)	-19.7	-36.1, -3.3	0.02	-17.3	-35.5, 1.0	0.06	-2.5	-27.0, 22.1	0.84	-0.06
Carbohydrates (g/day)	-39.6	-73.4, -5.7	0.02	-22.3	-59.9, 15.2	0.24	-17.2	-67.8, 33.4	0.51	-0.23
Saturated fat (g/day)	-9.3	-16.4, -2.2	0.01	-10.1	-17.9, -2.2	0.01	0.8	-9.8, 11.4	0.89	0.05
Sugar (g/day)	-19.1	-43.6, 5.4	0.13	-19.6	-46.7, 7.4	0.16	0.5	-36.0, 37.0	0.98	0.01
Added sugar (g/day)	-6.3	-21.4, 8.9	0.42	-11.7	-28.5, 5.1	0.17	5.4	-17.2, 28.0	0.64	0.16
Fibre (g/day)	0.8	-4.3, 6.0	0.75	3.1	-2.7, 8.8	0.30	-2.2	-10.0, 5.5	0.57	-0.12
Alcohol (g/day)	-0.72	− 1.2, -0.2	0.009	-0.5	-1.1, 0.1	0.09	-0.2	− 1.0, 0.6	0.64	-0.38
Fruit serves/day	0.1	-0.5, 0.7	0.75	0.6	-0.1, 1.2	0.08	-0.5	-1.4, 0.4	0.27	-0.40
Vegetable serves/day	0.3	-0.4, 0.9	0.48	0.8	0.0, 1.6	0.04	-0.6	-1.6, 0.5	0.29	-0.22
Seafood serves/week	0.6	0.0, 1.2	0.04	0.4	-0.2, 1.1	0.22	0.2	-0.7, 1.1	0.66	0.12
Dairy serves/day	0.0	-0.4, 0.5	0.89	-0.5	-1.0, 0.0	0.05	0.5	-0.1, 1.2	0.12	0.44
Grain serves/day	-0.3	-0.9, 0.2	0.25	0.1	-0.5, 0.8	0.68	-0.5	-1.3, 0.4	0.28	-0.36
Red meat serves/week	0.2	-1.4, 1.8	0.78	0.2	-1.6, 2.0	0.82	0.0	-2.4, 2.4	0.99	0.00
ANTHROPOMETRIC OUTCOMES										
Weight (kg)	-5.9	-8.0, -3.8	< 0.001	-6.4	-8.4, -4.4	< 0.001	0.5	-2.5, 3.4	0.76	0.03
Waist circumference (cm)	-8.9	-11.7, -6.1	< 0.001	-9.8	-12.5, -7.2	< 0.001	1.0	-2.9, 4.8	0.62	0.08
OTHER										
Patient Activation Measure	7.0	1.9, 12.1	0.01	6.9	1.5, 12.4	0.01	0.1	-7.4, 7.5	0.99	0.00
SF-12 Mental Score	-0.3	-1.7, 1.0	0.66	-1.4	-2.8, 0.0	0.05	1.1	-0.9, 3.0	0.28	0.59
SF-12 Physical Score	-0.3	-1.1, 0.4	0.38	0.2	-0.6, 1.0	0.58	-0.6	-1.7, 0.5	0.32	-0.43
SF-12 Total Score	-0.7	-2.2, 0.9	0.41	-1.0	-2.6, 0.6	0.21	0.4	-1.9, 2.6	0.74	0.17
KOOS Symptom Score	16.4	4.9, 27.9	0.01	11.0	2.4, 19.6	0.01	5.4	-9.0, 19.7	0.46	0.25
KOOS Pain Score	18.1	7.3, 28.9	0.001	8.6	0.5, 16.7	0.04	9.5	-4.0, 23.1	0.17	0.47
KOOS Function Score	25.2	12.6, 37.8	< 0.001	7.8	-1.6, 17.3	0.10	17.4	1.6, 33.1	0.03^a^	0.80
KOOS Sport Score	21.7	5.6, 37.7	0.001	14.5	2.4, 26.7	0.02	7.1	-13.0, 27.3	0.49	0 0.42
KOOS Quality of Life Score	21.5	9.3, 33.7	0.001	13.7	4.6, 22.9	0.003	7.7	-7.5, 23.0	0.32	0.40

a. After adjusting for multiple testing (Bonferroni adjusted *p*-value 0.002) this result was no longer significant

## Discussion

The current study aimed to assess feasibility, acceptability and preliminary efficacy on diet quality of adding MNT into the HWFL program. Overall, the addition of MNT into the HWFL program was shown to be highly accepted by participants, with high retention rates and low drop our rates indicating the intervention is feasible. Findings showed that both the usual care HWFL program and the HWFL + MNT resulted in significant improvements in diet quality, with no significant differences between group. However, for those with knee osteoarthritis, there were significant improvements in knee osteoarthritis outcomes in the HWFL + MNT intervention group compared to the usual care group, though these results did not remain significant after adjusting for multiple testing.

The addition of MNT to the HWFL program was highly acceptable to intervention participants, with 90% of participants reporting MNT was supportive and tailored to them as an individual. Current obesity guidelines support the need for personalised MNT to assist with weight management and long-term dietary behaviour change [43–45]. However, the personalisation of these interventions is important to ensure participant acceptability [46].Interventions that are more accepted have a greater likelihood of achieving positive changes in behaviour [47]. The current study was identified as both personalised and highly accepted by intervention participants, this confirms that the methods used (AES-Heart and PNQ) are appropriate for MNT intervention personalisation. Future interventions should consider comparing acceptability of HWFL program components between groups to be able to determine whether the addition on MNT resulted in greater acceptability of the HWFL overall. At baseline diet quality was poor. Similar to the general Australian population, participants within this study had approximately a third of energy intake coming from non-core foods [48]. Compared to Australian Nutrient Reference Values intakes of saturated fat (mean 36% energy) exceeded recommendations to limit saturated fat to < 10% energy [49] and is approximately three times higher than the estimated average intake for Australian adults [50]. Intake of added sugars was below recommendations from the World Health Organization of a maximum of 50 g per day [49]. Alcohol intake on average exceeded recommendations of a maximum of 10 standard drinks per week (~ 14 g alcohol/day) [51]. However, it is important to note that this data were skewed (range 0–78 g/day) with some participants reporting no alcohol intake and others having very high intakes. Average fibre intakes met recommendations [42] and while fruit and vegetable serves were higher than average Australian intakes [52] they did not meet national dietary recommendations of 2 serves of fruit and 5–6 serves of vegetables per day [40].

Both the intervention and usual care groups significantly improved their diet quality, however there were no significant between group differences identified. The HWFL program coaching provides nutrition information, practical resources and strategies to help participants lose weight [24]. Therefore, significant improvements in both the usual care and HWFL + MNT intervention arms were anticipated. Improvements in both the usual care and HWFL + MNT intervention arms for diet quality and weight may reflect behaviour change, with increased capability through nutrition education and self-monitoring tools, greater opportunity arising form the program structure, support and resources, and enhanced motivation from regular feedback and accountability. Further, the additional 1.5 h of MNT provided to participants in the intervention arm, focusing on addressing barriers and facilitators to dietary behaviour change, may have further strengthened opportunity as well as enhanced motivation, with regular dietary feedback and accountability to achieve goals prior to subsequent dietitian consults.

Interestingly, there were more within group improvements in dietary intake in the participants in the intervention arm, particularly with alcohol intake. Alcohol intake is associated with poorer health outcomes such as CVD, liver disease and some cancers [53], and is a risk factor for obesity [54]. There is limited evidence of the effects of MNT or individual lifestyle counselling on alcohol intake [55], but due to the personalisation of advice in the intervention arm this significant reduction may have been due to several participants in the intervention arm setting goals regarding reducing alcohol intake in their first consultation with the APD (data not presented). The intervention arm also reported significantly reduced total energy intakes, but this was not significant between groups. While a limitation of FFQs is that they are prone to systematic errors which affects accuracy of self-reported energy intake [56], this noticeable reduction in energy intake may have been due to significant reduction in fat intake, which a the most energy-dense macronutrient, in the intervention arm but not the usual care arm. Future studies in larger samples sizes are warranted to test this as there is a possibility of type 2 error occurring as this sample size may not be large enough to detect a significant between group difference in dietary outcomes.

Changes in anthropometric and patient activation measures both significantly improved in the intervention and usual care arms, but there were no between group differences. The addition of MNT did not significantly affect anthropometric outcomes. Prior studies have shown that a focus on diet quality, specifically increasing fruit and vegetable intakes in intervention studies may have little to no effect on weight loss [57, 58]. Therefore, it is promising to show that an additional focus on diet quality did not impact weight loss outcomes expected as part of the HWFL program. Similarly, both the HWFL and HWFL + MNT arms significantly improved patient activation and therefore increased participants engagement and self-management of their own health. Increased patient engagement in their management of their own health is an indicator of intervention success [21, 59] and improved long term management of health conditions [17, 18]. Therefore, given both the HWFL program and the addition of MNT can be defined as successful interventions that increase patient engagement in their health and healthcare outcomes, future studies should consider assessing the impact on longer term weight loss success.

While the MNT intervention was not specifically designed to focus on osteoarthritis outcomes, knee osteoarthritis outcomes significantly improved in the intervention group, particularly the KOOS function score which was significantly greater than the usual care arm at the end of the intervention. Dietary interventions, including those using partial VLEDs similar to the current study, have been shown to significantly reduce weight and improvements in KOOS function in people with osteoarthritis [60, 61]. A recent systematic review, identified favourable improvements in function when patients were counselled to increase diet quality through replacing refined grains and processed foods with increased intakes of plant-based foods and fish [62]. The MNT arm increased their intake of seafood and had marginally (but not significantly) greater improvements in diet quality compared to the usual care groups, which may explain why there was a significant improvement in function in the intervention arm but not the control arm. There is limited research on nutrition counselling and MNT in people with osteoarthritis. Further research is required to understand how MNT can be used to support patients with osteoarthritis to improve osteoarthritis outcomes and function. These results are promising and highlight the potentially important role for MNT in improving diet quality.

Recommendations for practice:

- Although only small differences were identified, up to four MNT consultations may be helpful to participants in the HWFL program to assist with personalising dietary recommendations. Participants who may benefit the most may be participants with higher baseline alcohol intake, osteoarthritis or those interested in receiving further support.
- Future studies could assess the optimal number of MNT consultations to be included alongside the HWFL program to optimise diet quality, health and wellbeing outcomes.
- In addition to current data collection tools within the HWFL program, consideration could be given to implementing the Australian Eating Survey as a cost-effective way to provide personalised dietary assessment and build on current care. This would assist with long term monitoring of diet quality in the changing landscape of weight management interventions, particularly with the greater usage of weight loss medications, for which there is limited knowledge of impact on actual diet quality [63].
- Long term follow-up post-HWFL intervention is recommended to identify how these interventions impact diet quality after the end of the intervention. This is particularly of interest considering prior research using the same methods of personalisation for MNT have shown that 6 and 12 months after intervention there was no relapse in diet quality [64].

There are several strengths and limitations of the current study. Strengths include that the method of dietary personalisation is informed by previous research [30, 34, 64] and the use of validated tools both within the HWFL program [36–38] and within the MNT intervention [29, 30]. Limitations include that this was a pilot project and therefore no sample size calculation was used which may limit the ability to identify between group differences. A retrospective sample size calculation was completed which would require a sample of 248 participants per group to detect a 5.9% difference in percentage energy from core food [64], using a standard deviation of 33% (from the current study), at 80% power and significance set at 0.05. Future studies, considering using a health-related biomarker, such as HbA1c, could use a smaller sample of 54 participants per group, based on detecting a between group difference of 0.2% (SD 4%) [64]. Further, only self-reported assessments were used for all outcomes, which are at risk of bias including misreporting and social desirability biases. Future work could consider including objective measures of health and dietary intake, including nutrition and chronic disease biomarkers. In addition, selection bias may have occurred in this study as participants opting into an existing commercial program is likely to represent a motivated subset, as well as healthy user bias, where healthier or health-conscious individuals are more likely to participate in research [65]. Whilst this study utilised a validated food frequency questionnaire (FFQ), the AES-Heart, a limitation of FFQs is that they use a defined and hence limited food list, are semi-quantitative and are affected by recall bias [66]. Weight data may also have been affected by self-report bias. Moreover, the majority of participants were older adults with osteoarthritis and therefore the results may not be generalisable to all four HWFL programs. Due to the short term follow up period of 18 weeks, we are unable to draw conclusion regarding long term maintenance. Another limitation is the absence of a control for the dietitian contact time, with participants in the intervention group receiving an additional 1.5 h of support. This may have contributed to the participants perceived benefits. Therefore, future studies exploring the addition of MNT should consider matching contact time between groups. Lastly, the study did not measure or analyse behaviour objectively, which limits the ability to understand how or why improvements occurred. Future studies in this area should consider measuring and analysing behaviour objectively, so conclusions can be drawn. The integration of MNT, facilitated by APDs, into established telehealth programs invites practice relevance, increasing access to MNT for individuals living with chronic conditions and overweight or obesity. Future trials should consider behavioural mechanisms including self-efficacy and goal setting, as well as objectively measuring long term behaviour change and adherence to dietary recommendations to further understanding of how MNT can influence patient behaviour and health outcomes. This will support the development of a larger, acceptable dietary behaviour interventions that optimises health outcomes for those living with chronic conditions, including overweight and obesity.

## Conclusion

The current pilot study identified that both the HWFL and HWFL + MNT arms significantly improved diet quality while subsequently achieving statistically significant weight loss, with no between group differences. The HWFL + MNT arm resulted in significant improvements in knee osteoarthritis function compared to the usual care arm, however caution with this finding should be considered as this result did not remain statistically significant after adjusting for multiple testing. No other between group differences were reported. Overall, acceptance of the HWFL + MNT intervention was high with participants reporting that the methods used for personalisation were highly accepted. Future longer-term, adequately powered studies are required to assess the impact of the HWFL program and HWFL + MNT on diet quality after the cessation of the intervention.

## Supplementary Information

Supplementary Material 1.

## Data Availability

Data is available upon reasonable request by contacting the corresponding author.
